# A retrospective study of the incidence, clinical characteristics, identification, and antimicrobial susceptibility of bacteremic isolates of *Acinetobacter ursingii*

**DOI:** 10.1186/s12879-015-1145-z

**Published:** 2015-09-30

**Authors:** Chun-Hsiang Chiu, Yi-Tzu Lee, Yung-Chih Wang, Ti Yin, Shu-Chen Kuo, Ya-Sung Yang, Te-Li Chen, Jung-Chung Lin, Fu-Der Wang, Chang-Phone Fung

**Affiliations:** Division of Infectious Diseases and Tropical Medicine, Department of Internal Medicine, Tri-Service General Hospital, National Defense Medical Center, Taipei, Taiwan; Institute of Clinical Medicine, School of Medicine, National Yang-Ming University, No.155, Sec.2, Linong Street, Taipei 112 Taiwan, Republic of China; Emergency Department, Taipei Veterans General Hospital, Taipei, Taiwan; Department of Nursing, Tri-Service General Hospital, National Defense Medical Center, Taipei, Taiwan; School of Nursing, National Yang-Ming University, Taipei, Taiwan; National Institute of Infectious Diseases and Vaccinology, National Health Research Institutes, Zhunan, Miaoli County Taiwan

**Keywords:** *Acinetobacter ursingii*, Clinical characteristics, Identification, Minimal inhibitory concentration, Bacteremia

## Abstract

**Background:**

*Acinetobacter ursingii* bacteremia is rarely reported. We investigated the incidence and clinical features of *A. ursingii* bacteremia, performance of the identification system, and antimicrobial susceptibility of the isolates. *Acinetobacter ursingii* bacteremia patients were compared with *A. baumannii* bacteremia patients.

**Methods:**

In this 9-year retrospective study, *A. ursingii* was identified using 16S rRNA and 16S–23S rRNA internal transcribed spacer sequence analysis. The performances of the Vitek 2, Phoenix, and matrix-assisted laser desorption ionization time-of-flight (MALDI-TOF) mass spectrometer systems for identifying isolates were tested. Pulsed-field gel electrophoresis (PFGE) was used to determine the clonality of the isolates. The minimal inhibitory concentrations of the antimicrobials were determined using the Vitek 2 system.

**Results:**

Nineteen patients were identified. *Acinetobacter ursingii* was noted in 1.5–5.2 % of all *Acinetobacter* bacteremia cases. For the PFGE analysis, two isolates had smeared DNA, two had 93 % similarity, and 15 had similarity <80 %. Among 16 patients with complete medical records, 10 (62.5 %) had no identifiable source of *A. ursingii* bacteremia. Most patients (*n* = 12) had underlying malignant disease. Patients with *A. ursingii* bacteremia had lower Acute Physiology and Chronic Health Evaluation II scores than those with *A. baumannii* bacteremia (median [interquartile range], 17.1 [10.0–24.7] vs. 24.9 [14.6–35.1]). Patients with *A. ursingii* bacteremia were also less likely admitted to the intensive care unit than patients with *A. baumannii* bacteremia (18.8 % vs 63.5 %, *p* value < 0.01). About half of the patients with *A. ursingii* (50.8 %) and *A. baumannii* bacteremia (62.5 %) had received inappropriate antimicrobial therapy within 48 h after bacteremia onset. However, patients with *A. ursingii* bacteremia had significantly lower 14-day (6.25 % vs 29.8 %, *p* value = 0.04) and 28-day mortality rates (6.25 % vs 37.3 %, *p* value = 0.02) than patients with *A. baumannii* bacteremia. Nine isolates (47.4 %) were correctly identified as *A. ursingii* and the other 10 isolates (52.6 %) were incorrectly identified as *A. lwoffii* by the Vitek 2 system. The Phoenix system incorrectly identified all 19 isolates. The MALDI-TOF mass spectrometer system correctly identified all 19 isolates. All the *A. ursingii* isolates were resistant or showed intermediate susceptibility to ceftriaxone and ceftazidime, but were susceptible to levofloxacin and imipenem.

**Conclusions:**

*Acinetobacter ursingii* is a rare pathogen that mostly caused primary bacteremia in patients with malignancies. Patients with *A. ursingii* bacteremia had significantly lower disease severity and mortality rates than patients with *A. baumannii* bacteremia.

## Background

The genus *Acinetobacter* comprises a heterogeneous group of non-motile, aerobic, oxidase negative, non-fermentative, gram-negative coccobacilli [1, 2]. They are widespread in natural moist and hospital environments, and are associated with skin colonization of hospitalized patients [3]. Although they were thought to have low pathogenicity, the *Acinetobacter* species have been recognized as opportunistic nosocomial pathogens that mainly affect immune-compromised patients and patients hospitalized in intensive care units (ICUs) [4]. It has emerged as one of the most troublesome pathogens for health care institutions globally over the past 2 decades, owing to its increasing prevalence and rapid development of drug resistance.

The genus *Acinetobacter* comprises 39 genomic species (http://www.bacterio.net/acinetobacter.html) [5]. While *Acinetobacter* species such as *A baumannii*, *A. nosocomialis*, and *A. pittii* are frequently isolated as human pathogens [6–11]; other species, such as *A. ursingii,* are rarely reported as pathogens [12, 13]. The low incidence of *A. ursingii* infection may be further complicated by the inaccurate identification tools used in clinical laboratories. In this study, we aimed to describe the incidence and clinical characteristics of *A. ursingii* bacteremia, the performance of two phenotypic identification systems and one matrix-assisted laser desorption ionization time-of-flight (MALDI-TOF) mass spectrometer, and the antimicrobial susceptibilities of the isolates. Owing to the predominance of *A. baumannii* in clinical settings, we also compared the clinical features of *A. ursingii* and *A. baumannii* bacteremia.

## Methods

### Subjects

Patients who were admitted to the Taipei Veterans General Hospital (T-VGH) from January 2000 to December 2008, were included. T-VGH is a 2980-bed medical center that serves about 120 thousand person-times pear year. It serves not only veterans but also their families and other individuals. The charts were reviewed from all patients with symptoms and signs of infection who had at least one positive blood culture for *A. ursingii* and *A. baumannii*. If patients had two or more positive blood cultures, only the first blood culture was included. The source of infection was determined as recommended by the Centers of Disease Control guidelines [14, 15]. Patients under 18 years of age and those with incomplete medical records were excluded. The protocol was approved by the T-VGH Institutional Review Board (approval number: 2011-10-012IC), with a waiver for informed consent.

### Data collection

Medical records were reviewed to obtain clinical information, including demographic characteristics; underlying diseases; severity of illness; the presence of a ventilator, central venous catheters, a nasogastric tube, or a Foley catheter at the time of onset of bacteremia; intensive care unit (ICU) hospitalization; and survival. Chronic kidney disease was defined as an estimated glomerular filtration rate <60 mL/min/1.73 m^2^. Neutropenia was defined as an absolute neutrophil count of <0.5 × 10^9^ neutrophils/L. Recent surgery was defined as any operation performed within 4 weeks prior to the onset of bacteremia. Shock was defined as hypotension (systolic blood pressure [SBP] <90 mmHg, mean arterial pressure <70 mmHg, or a SBP decrease > 40 mmHg) with evidence of end organ dysfunction. Bacteremia cases without a definite identified source were defined as primary bacteremia. The severity of illness was evaluated using the Acute Physiology and Chronic Health Evaluation II (APACHE II) score [16] within 24 h prior to bacteremia onset.

Appropriate antimicrobial therapy was defined as administration of at least one antimicrobial agent to which the causative pathogen was susceptible within 48 h of the onset of bacteremia by an approved route and at a dosage consistent with end organ(s) function. Antimicrobial therapy that did not meet this definition was considered inappropriate. Monotherapy with an aminoglycoside was not considered the appropriate therapy. All-cause 14-day and 28-day mortality rates were recorded.

### Bacterial isolates, genotypic and phenotypic identification, pulsed-field gel electrophoresis analysis, and determination of antimicrobial minimal inhibitory concentration

From January 2000 to December 2008, 616 clinical isolates of *Acinetobacter* were isolated from blood samples at T-VGH. All isolates were presumed to be *Acinetobacter* species, as determined using phenotypic methods with the 32GN system or the Vitek 2 system (bioMérieux, Marcy l’Etoile, France). These isolates were included in our study for further identification. A multiplex-polymerase chain reaction method was then used to identify *A. baumannii* at the genomic species level [17]. Isolates belonging to *non-A. baumannii* species were identified as *A. ursingii* using 16S rRNA gene sequence [18] and confirmed by 16S-23S rRNA internal transcribed spacer (ITS) sequence analysis [19]. Pulsed-field gel electrophoresis (PFGE) was performed to determine the clonality of the isolates [20]. These *A. ursingii* isolates were then used to determine the performance of the Vitek 2 (bioMérieux), Phoenix (Becton Dickinson, NJ, USA), and matrix-assisted laser desorption ionization time-of-flight (MALDI-TOF) mass spectrometer systems (Bruker Daltonics, Billerica, MA) in the identification of this species. The antimicrobial minimal inhibitory concentrations (MICs) for the isolates were determined by using the Vitek 2 system (bioMérieux). The tested antimicrobials were ampicillin-sulbactam, ceftazidime, ceftriaxone, cefepime, imipenem, amikacin, gentamicin, ciprofloxacin, levofloxacin, and colistin. The breakpoint interpretation was determined according to the recommendations of the Clinical Laboratory Standards Institute (CLSI) [21].

### Statistical analysis

To assess differences, the Student’s *t*-test or the Mann–Whitney rank sum test was used to analyze continuous variables, while the chi-square test with Yate’s correction or Fisher’s exact test was used to compare discrete variables. Time to mortality was analyzed using the Kaplan–Meier survival analysis and the long-rank test. A *p*-value <0.05 was considered statistically significant. All analyses were processed with the Statistical Package for the Social Sciences (SPSS) software version 18.0 (SPSS, Chicago, IL, USA).

## Results

### Incidence and clinical features of *A. ursingii* bacteremia

**Fig. 1:**
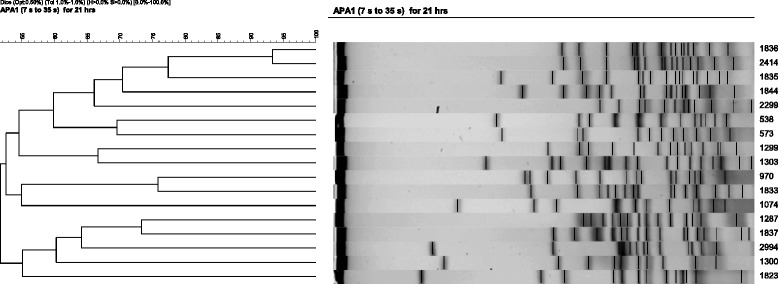
Pulse-field gel electrophoresis patterns of the *Acinetobacter ursingii* isolates

During the study period, 616 patients were found to have *Acinetobacter* species bacteremia and were included in our study. Among the isolates, 19 (3.1 %) were identified as *A. ursingii* by16S rRNA gene sequence analysis and confirmed by ITS sequence analysis (similarity: 98-99 % to reference strains) and 252 (40.9 %) as *A. baumannii*. For the PFGE analysis, two isolates had smeared DNA, two had 93 % similarity, and 15 had similarity less than 80 % (Fig. 1). The annual incidence of *A. ursingii* among *Acinetobacter* species bacteremia in this study was 1.5–5.2 %.

The clinical data of the first three *A. ursingii* bacteremia patients were incomplete and they were therefore excluded from further analysis. The comparison of demographic features, underlying diseases, APACHE II scores, and outcomes of *A. ursingii* and *A. baumannii* bacteremia patients included in this study are summarized in Table 1.

**Table 1:** Demographic data, clinical features, and outcomes of patients with *Acinetobacter ursingii* and *Acinetobacter baumannii* bacteremia

	*Acinetobacter ursingii* (*n* = 16)	*Acinetobacter baumannii* (*n* = 252)	*p* value
	n (%)/median (Q1-Q3)/mean ± S.D.		
Gender, male	7 (43.8 %)	183 (72.6 %)	0.01
Age in years (median, IQR)	66.6 (50.0–83.2)	68.7 (52.6–84.9)	0.61
Source			
Respiratory tract	4 (25 %)	130 (51.6 %)	0.04
Intra-abdominal	1 (6.25 %)	18 (7.1 %)	0.89
Urinary tract	0	19 (7.5 %)	0.25
Intravenous device	0	12 (4.8 %)	0.37
Wound	0	10 (4.0 %)	0.42
Other	1 (6.25 %)	13 (5.2 %)	0.85
Unknown	10 (62.5 %)	50 (19.8 %)	<0.01
Comorbidity			
Diabetes mellitus	2 (12.5 %)	66 (26.2 %)	0.22
Hypertension	7 (43.8 %)	78 (31.0 %)	0.29
Coronary artery disease	2 (12.5 %)	30 (11.9 %)	0.94
Congestive heart failure	3 (18.8 %)	21 (8.3 %)	0.16
Chronic obstructive pulmonary disease	2 (12.5 %)	40 (15.9 %)	0.72
Cerebral vascular disease	2 (12.5 %)	47 (18.7 %)	0.54
Chronic kidney disease	3 (18.8 %)	47 (18.7 %)	0.99
End stage renal disease	1 (6.3 %)	11 (4.4 %)	0.72
Alcoholism	1 (6.3 %)	22 (8.7 %)	0.73
Malignancy	12 (75 %)	88 (34.9 %)	<0.01
Solid malignancy	7 (43.8 %)	69 (27.4 %)	0.16
Hematologic malignancy	5 (31.3 %)	19 (7.5 %)	<0.01
Neutropenia	4 (25.0 %)	9 (3.6 %)	<0.01
Trauma	0	8 (3.2 %)	0.47
Surgery in 1 month	4 (25 %)	88 (34.92 %)	0.42
Procedure			
Ventilator	4 (25 %)	137 (54.4 %)	0.02
Endotracheal tube or tracheostomy	4 (25 %)	187 (74.2 %)	<0.01
Central venous catheter	6 (37.5 %)	130(51.6 %)	0.27
Artery line	6 (37.5 %)	52 (20.6 %	0.26
Foley catheter	6 (37.5 %)	157 (62.3 %)	0.05
Nasogastric tube	6 (37.5 %)	180 (71.4 %)	<0.01
Thoracic drain	0	9 (3.6 %)	0.44
Hemodialysis	1 (6.25 %)	16 (6.35 %)	0.99
Total parental nutrition	1 (6.25 %)	24 (9.5 %)	0.66
Other			
Chemotherapy	9 (56.3 %)	28 (11.1 %)	<0.01
Steroid use	3 (18.8 %)	69 (27.4 %)	0.45
Shock	2 (12.5 %)	54 (21.4 %)	0.39
Acquired in ICU	3 (18.8 %)	160 (63.5 %)	<0.01
APACHE II score (median, IQR)	17.1 (10.0–24.7)	24.9 (14.6–35.1)	<0.01
Appropriate antimicrobial therapy	10 (62.5 %)	128 (50.8 %)	0.36
Hospitalized days (median, IQR)	28 (13–60)	39 (18–73.5)	0.56
Mortality			
14-day mortality	1 (6.25 %)	75 (29.8 %)	0.04
28-day mortality	1 (6.25 %)	94 (37.3 %)	0.02

The data were presented in number and percentage, unless indicated otherwise. *IQR* interquartile range, *ICU* intensive care unit, *APACHE II* Acute Physiology and Chronic Health Evaluation II

The gender of the patients with *A. ursingii* bacteremia was similar, while most patients with *A. baumannii* bacteremia were male. Primary bacteremia was mostly noted among those with *A. ursingii* infection (62.5 %), while respiratory tract infection (51.6 %) was the major source of *A. baumannii* bacteremia. The comorbidity of these two groups was similar, except that *A. ursingii* bacteremia tended to occur in patients with hematologic malignancies (*p* value < 0.01) or neutropenia who had undergone chemotherapy in the past month (*p* value <0.01). Patients with *A. ursingii* bacteremia had lower APACHE II scores (*p* value < 0.01), and less often acquired infection in the intensive care unit than patients with *A. baumannii* bacteremia (*p* value < 0.01). Consequently, patients with *A. ursingii* bacteremia underwent fewer invasive produces, including endotracheal tubing or tracheostomy (*p* value < 0.01), nasogastric tubing (*p* value < 0.01), and ventilator support (*p* value = 0.02).

**Fig. 2:**
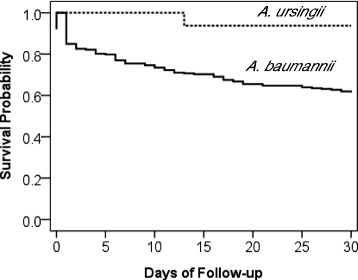
The Kaplan-Meier survival curves of patients with bacteremia caused by *Acinetobacter ursingii* and *Acinetobacter baumannii*. The 30-day mortality rate of *A. ursingii* bacteremia was significantly lower than that of *A. baumannii* bacteremia (*p*-value = 0.0352)

About half of the patients with *A. ursingii* (50.8 %) and *A. baumannii* bacteremia (62.5 %) had received inappropriate antimicrobial therapy within 48 h of the onset of bacteremia. However, the 14-day (*p* value = 0.04) and 28-day (*p* value = 0.02) mortality rates of the *A. ursingii* group were significantly lower than those of the *A. baumannii* group. The Kaplan-Meier survival curves also showed that patients with *A. ursingii* had a higher cumulative survival rate than those with *A. baumannii* (Fig. 2).

### Identification

The identifications of these 19 clinical isolates of *A. ursingii* under the Vitek 2, Phoenix, and MALDI-TOF mass spectrometer systems are listed in Table 2. According to the ID-GNB card of the Vitek 2 system, 9 isolates (47.4 %) were correctly identified as *A. ursingii.* The other 10 isolates (52.6 %) were incorrectly identified as *A. lwoffii.* The Phoenix system incorrectly identified all 19 isolates. Among them, 15 isolates (78.9 %) were misidentified as *Alcaligenes faecalis*, 3 isolates (15.8 %) as *A. lwoffii/haemolyticus*, and 1 isolate (5.3 %) as *Moraxella* species. All the 19 isolates (100 %) were correctly identified as *A. ursingii* by using MALDI-TOF mass spectrometer analysis.

**Table 2:** Identifications obtained with the Phoenix, Vitek 2 systems, and matrix-assisted laser desorption ionization time-of-flight mass spectrometer for the *Acinetobacter ursingii* isolates

No.	Phoenix (confidence value)	VITEK 2 ID-GNB card (confidence value)	MALDI-TOF (confidence value)
1	Moraxella species (97 %)	*Acinetobacter lwoffii* (94 %)	*Acinetobacter ursingii* (99.9 %)
2	*Acinetobacter lwoffii*/*haemilyticus* (90 %)	*Acinetobacter lwoffii* (94 %)	*Acinetobacter ursingii* (99.9 %)
3	*Alcaligenes faecalis* (95 %)	*Acinetobacter ursingii* (98 %)	*Acinetobacter ursingii* (99.9 %)
4	*Alcaligenes faecalis* (95 %)	*Acinetobacter ursingii* (97 %)	*Acinetobacter ursingii* (99.9 %)
5	*Acinetobacter lwoffii*/*haemolyticus* (90 %)	*Acinetobacter lwoffii* (97 %)	*Acinetobacter ursingii* (99.9 %)
6	*Alcaligenes faecalis* (90 %)	*Acinetobacter lwoffii* (95 %)	*Acinetobacter ursingii* (99.9 %)
7	*Alcaligenes faecalis* (95 %)	*Acinetobacter ursingii* (93 %)	*Acinetobacter ursingii* (99.9 %)
8	*Alcaligenes faecalis* (90 %)	*Acinetobacter ursingii* (93 %)	*Acinetobacter ursingii* (99.9 %)
9	*Alcaligenes faecalis* (96 %)	*Acinetobacter ursingii* (93 %)	*Acinetobacter ursingii* (99.9 %)
10	*Alcaligenes faecalis* (98 %)	*Acinetobacter ursingii* (94 %)	*Acinetobacter ursingii* (99.9 %)
11	*Alcaligenes faecalis* (98 %)	*Acinetobacter lwoffii* (91 %)	*Acinetobacter ursingii* (99.9 %)
12	*Acinetobacter lwoffii*/*haemolyticus* (90 %)	*Acinetobacter lwoffii* (93 %)	*Acinetobacter ursingii* (99.9 %)
13	*Alcaligenes faecalis* (98 %)	*Acinetobacter lwoffii* (91 %)	*Acinetobacter ursingii* (99.9 %)
14	*Alcaligenes faecalis* (98 %)	*Acinetobacter lwoffii* (90 %)	*Acinetobacter ursingii* (99.9 %)
15	*Alcaligenes faecalis* (90 %)	*Acinetobacter lwoffii* (95 %)	*Acinetobacter ursingii* (99.9 %)
16	*Alcaligenes faecalis* (95 %)	*Acinetobacter ursingii* (96 %)	*Acinetobacter ursingii* (99.9 %)
17	*Alcaligenes faecalis* (98 %)	*Acinetobacter ursingii* (93 %)	*Acinetobacter ursingii* (99.9 %)
18	*Alcaligenes faecalis* (95 %)	*Acinetobacter ursingii* (94 %)	*Acinetobacter ursingii* (99.9 %)
19	*Alcaligenes faecalis* (95 %)	*Acinetobacter lwoffii* (95 %)	*Acinetobacter ursingii* (99.9 %)

### Antimicrobial susceptibility

The antimicrobial susceptibility results of the 19 isolates are summarized in Table 3. All the *A. ursingii* isolates were resistant or had intermediate susceptibility to ceftriaxone and ceftazidime, and all were susceptible to levofloxacin and imipenem. About half of the *A. ursingii* isolates were resistant or had intermediate susceptibility to ciprofloxacin (47.4 %) and cefepime (42.1 %). A small number of the isolates were resistant or had intermediate susceptibility to amikacin (10.5 %), gentamicin (15.8 %), ampicillin-sulbactam (21.1 %), and colistin (15.8 %).

**Table 3:** Antimicrobial susceptibility profiles of the 19 *Acinetobacter ursingii* isolates

	No. of *A. ursingii* isolates						
	S	I	R	I + R	MIC range	MIC50	MIC90
Ampicillin-Sulbactam	15	3	1	21.1 %	≤2–16	≤2	16
Ceftazidime	0	3	16	100.0 %	16– ≥ 64	≥64	≥64
Ceftriaxone	0	10	9	100.0 %	16– ≥ 64	32	≥64
Cefepime	11	0	8	42.1 %	4–32	8	32
Imipenem	19	0	0	0.0 %	≤0.25	≤0.25	≤0.25
Amikacin	17	0	2	10.5 %	≤2– ≥ 64	≤2	≤2
Gentamicin	16	1	2	15.8 %	≤1– ≥ 16	≤1	8
Ciprofloxacin	10	7	2	47.4 %	≤0.25– ≥ 4	1	2
Levofloxacin	19	0	0	0.0 %	≤0.12–1	0.5	1
Colistin	16	0	3	15.8 %	≤0.5–4	0.5	4

*S* susceptible, *I* intermediate susceptible, *R* resistant

Minimal inhibitory concentrations (MICs) are given in milligrams per liter (mg/L)

## Discussion

*Acinetobacter ursingii* is a rare pathogen that mostly causes bacteremia in patients with hematologic malignancies. In this study, most cases were of primary bacteremia, and patients had milder disease severity and underwent fewer invasive procedures than patients with *A. baumannii* bacteremia. Although more than half of the patients with *A. ursingii* and *A. baumannii* bacteremia had undergone inappropriate antimicrobial therapy within 48 h of the onset of bacteremia, the 14-day and 28-day mortality rates of patients with *A. ursingii* bacteremia were significantly lower than those of patients with *A. baumannii* bacteremia.

As in previous studies [12, 22], a low incidence of *A. ursingii* bacteremia was noted in our study (1.5–5.2 % during the study period). Compared to the risk factors and clinical characteristics of patients with *A. baumannii* bacteremia, patients with *A. ursingii* bacteremia are believed to be more immunosuppressed than patients with *A. baumannii* bacteremia, due to the higher concurrence rate in patients with hematologic malignancy, neutropenia, and chemotherapy treatment. Compared to the condition of patients with *A. baumannii* bacteremia*,* that of patients with *A. ursingii* seemed less severe, as indicated by lower APACHE II scores, fewer ICU admissions and invasive procedures, and lower mortality rates. The results indicated a lower virulence of *A. ursingii*, and this may account for the lower incidence of *A. ursingii* bacteremia.

It is unclear why most of the *A. ursingii* cases were primary bacteremia without an obvious source of infection. Among the patients with *A. ursingii* bacteremia, central venous catheters were placed in 6 patients (37.5 %), arterial catheters in 6 patients (37.5 %), and total parenteral nutrition in 1 patient (6.25 %). One patient (6.25 %) required dialysis and 4 patients (25 %) needed ventilator support at the onset of bacteremia. The intravascular device may serve as a port of entry for *A. ursingii* bacteremia.

Phenotypic schemes are generally insufficient to accurately identify the *Acinetobacter* isolates at the species level [23–25]. Phenotypic identification by commercial colorimetric systems is also unsatisfactory [6, 12, 26, 27]. Using systems such as the Vitek 2, API20NE systems (bioMérieux, Marcy l’Etoile, France) and the Phoenix system, the clinically relevant species of the *A. calcoaceticus–A. baumannii* complex are frequently uniformly identified as *A. baumannii,* and many other species are not identified [6, 12, 26, 27]. On comparison, the Vitek 2 systems in our study could correctly identify about half of the isolates, and Phoenix systems failed to correctly identify any. Protein fingerprinting using a MALDI-TOF mass spectrometer is a promising molecular method for rapid identification of *Acinetobacter* species with high-throughput capability. A previous study revealed a 72.4 % correct identification rate for *Acinetobacter* species by using MALDI-TOF mass spectrometer [28]. The correct rate of *A. ursingii* identification was 93.3 % in that study. As the database of the MALDI-TOF mass spectrometer expanded, 100 % of the *A. ursingii* isolates were correctly identified in our study.

In our study, all the *A. ursingii* isolates exhibited high resistance to third- and fourth-generation cephalosporins. These results suggest the presence of some extended spectrum β-lactamase, which deserves further study. More than 80 % of the *A. ursingii* isolates were susceptible to imipenem, levofloxacin, amikacin, gentamicin, and colistin. While comparing the antimicrobial susceptibility of the *A. ursingii* isolates in our study with the isolates reported by Cattoir in 2006 [12], the resistance rate to ciprofloxacin, gentamicin, amikacin, and colistin seemed higher. Further studies are needed to elucidate the mechanisms underlying antimicrobial resistance.

## Conclusion

In conclusion, *A. ursingii* is a rare pathogen with a low mortality rate. This pathogen mostly causes primary bacteremia in patients with malignancies.
